# Quantitative promoter methylation analysis of multiple cancer-related genes in renal cell tumors

**DOI:** 10.1186/1471-2407-7-133

**Published:** 2007-07-23

**Authors:** Vera L Costa, Rui Henrique, Franclim R Ribeiro, Mafalda Pinto, Jorge Oliveira, Francisco Lobo, Manuel R Teixeira, Carmen Jerónimo

**Affiliations:** 1 Department of Genetics, Portuguese Oncology Institute, Porto, Portugal; 2 Department of Pathology, Portuguese Oncology Institute, Porto, Portugal; 3 Department of Urology, Portuguese Oncology Institute, Porto, Portugal; 4 Department of Pathology and Molecular Immunology, Institute of Biomedical Sciences Abel Salazar (ICBAS), University of Porto, Porto, Portugal; 5 School of Health Sciences, Fernando Pessoa University, Porto, Portugal

## Abstract

**Background:**

Aberrant promoter hypermethylation of cancer-associated genes occurs frequently during carcinogenesis and may serve as a cancer biomarker. In this study we aimed at defining a quantitative gene promoter methylation panel that might identify the most prevalent types of renal cell tumors.

**Methods:**

A panel of 18 gene promoters was assessed by quantitative methylation-specific PCR (QMSP) in 85 primarily resected renal tumors representing the four major histologic subtypes (52 clear cell (ccRCC), 13 papillary (pRCC), 10 chromophobe (chRCC), and 10 oncocytomas) and 62 paired normal tissue samples. After genomic DNA isolation and sodium bisulfite modification, methylation levels were determined and correlated with standard clinicopathological parameters.

**Results:**

Significant differences in methylation levels among the four subtypes of renal tumors were found for *CDH1 *(*p *= 0.0007), *PTGS2 *(*p *= 0.002), and *RASSF1A *(*p *= 0.0001). *CDH1 *hypermethylation levels were significantly higher in ccRCC compared to chRCC and oncocytoma (*p *= 0.00016 and *p *= 0.0034, respectively), whereas *PTGS2 *methylation levels were significantly higher in ccRCC compared to pRCC (*p *= 0.004). *RASSF1A *methylation levels were significantly higher in pRCC than in normal tissue (*p *= 0.035). In pRCC, *CDH1 *and *RASSF1A *methylation levels were inversely correlated with tumor stage (*p *= 0.031) and nuclear grade (*p *= 0.022), respectively.

**Conclusion:**

The major subtypes of renal epithelial neoplasms display differential aberrant *CDH1*, *PTGS2*, and *RASSF1A *promoter methylation levels. This gene panel might contribute to a more accurate discrimination among common renal tumors, improving preoperative assessment and therapeutic decision-making in patients harboring suspicious renal masses.

## Background

Cancer is currently a major cause of morbidity and mortality in Western World, only surpassed by cardiovascular diseases [1]. Kidney cancer accounts for approximately three percent of all solid neoplasms and its incidence appears to be rising [2]. Overall, it is estimated that 208 480 new cases of kidney cancer were diagnosed worldwide in 2002, with a total of 101 895 deaths [1]. In the USA, the adjusted incidence and mortality of this disease were approximately two times higher in males than in females, standing as the 7^th ^leading malignant condition among men and the 12^th ^among women [2]. In 2006, 38 890 new cases and 12 840 deaths are predicted to occur in the USA [2], whereas in Europe there were 85 719 cases and 45 270 deaths in 2002 [1]. The vast majority of renal cell tumors are sporadic as only 2% of cases are associated with inherited syndromes [3].

Renal cell tumors account for about 85 percent of all adult renal neoplasms, comprising a heterogeneous class of epithelial tumors that range in biological potential from entirely benign to highly malignant [4]. Common histopathologic subtypes include clear cell renal cell carcinoma (ccRCC, ~75% of surgically removed renal cell tumors), papillary RCC (pRCC, ~10%), chromophobe RCC (chRCC, ~5%), and oncocytoma (~5%) [5]. Most renal cell tumors are clinically silent in their earlier stages and 20–30% are diagnosed when metastatic spread has already occurred [6]. However, the widespread use of imagiologic testing (mainly ultrasonography) has increased the detection of renal masses, prompting new pre-operative diagnostic challenges as histological diagnosis using needle biopsy material meets with important limitations, hampering an accurate categorization [7]. Thus, there is a need for the development of new strategies both for early detection and differential diagnosis of renal cell tumors.

Epigenetic alterations and gene promoter hypermethylation in particular, provide an emerging class of cancer biomarkers, holding the promise of sensitive and accurate disease detection even at the earliest stages [8]. The power of epigenetic markers for molecular detection of the most common urological malignancy (*i.e.*, prostate cancer) has been demonstrated in previous studies from our research team and others [9-15]. Some previous studies attempted a characterization of the RCC methylome, but either they did not comprise some the most frequent histological subtypes [16-18], did not use the more sensitive and specific quantitative assays [18-20], or have examined only a limited number of primary RCC [16-18]. Thus, it might be stated that the discovery of methylation markers in RCC provides an attractive and still largely unexplored field for biomedical research.

In the present study we aimed at the definition of a gene promoter methylation panel that might discriminate among most prevalent types of epithelial kidney tumors, including the most common RCC subtypes and oncocytoma, which may serve as ancillary tools for diagnosis and prognosis assessment. For that purpose, the promoter region of 18 cancer-related genes (which were chosen based on their established relevance to various human cancers and, specifically, RCC), was surveyed for CpG methylation using quantitative real-time PCR in a relatively large series of kidney tumors and morphologically normal tissue. In addition, methylation data was correlated with the relevant clinicopathologic data.

## Methods

### Patients, sample collection, and DNA extraction

Eighty-five patients with ccRCC, pRCC, chRCC, or oncocytoma, consecutively diagnosed and treated with partial or radical nephrectomy at the Portuguese Oncology Institute – Porto, Portugal, between April 2001 and June 2003, were selected for this study after written informed consent was obtained. Samples of tumor and morphologically normal tissue distant to the primary tumor (when available) were immediately obtained after surgical resection, fresh-frozen, and stored at -80°C for further analysis. Additional samples were taken for routine pathological evaluation, after formalin-fixation and paraffin embedding. The correspondent hematoxylin-eosin-stained sections were examined by the same pathologist (RH) to determine the tumor type [4], nuclear grade [21], and pathological stage [4]. Frozen sections (five-micron thick) were cut and stained for the identification of the areas of tumor and morphologically normal tissue. Then, the tissue block was trimmed to maximize the yield of target cells (>70% of target cells). Subsequently, approximately fifty 12 μm thick sections were cut and every 5^th ^section was stained to ensure a uniform percentage of target cells and to exclude contamination of normal tissue samples with neoplastic cells. Genomic DNA was extracted from tumor and normal tissue using a standard technique comprising overnight digestion with proteinase K (20 mg/mL) in the presence of 10% SDS at 55°C, followed by phenol-chloroform extraction and precipitation with 100% ethanol [22].

Relevant clinical data was collected from patient's clinical records. These studies were approved by the institutional review board (IRB) of the Portuguese Oncology Institute – Porto.

### Bisulfite treatment and QMSP

Sodium bisulfite conversion of unmethylated (but not methylated) cytosine residues to uracil was performed as previously described [23]. Briefly, four μg of genomic DNA were denatured in 0.3 M NaOH for 20 min at 50°C. The denatured DNA was diluted in 450 μl of a freshly prepared solution of 125 mM hydroquinone and 2.5 M sodium bisulfite and was incubated for 3 h at 70°C. After incubation, modified DNA samples were desalted and purified through a column (Wizard DNA Clean-Up System; Promega, Madison, WI), treated again with sodium hydroxide for 10 min at room temperature, precipitated with 100% ethanol, resuspended in 240 μl of water, and stored at -80°C.

The chemically modified DNA was then used as a template for quantitative methylation-specific PCR [24]. In brief, primers and probes were designed to specifically amplify fully methylated bisulfite-converted complementary sequences of the promoter of interest. The 18 cancer-related genes used in this renal cancer detection panel were genes involved in cell communication and signal transduction (*APC *[25], *ARHI *[26], *CDH1 *[25], *CTNNB1 *[25], *SFN *[27]); cell cycle regulation (*ARF/p14 *[25], *CDKN2A/p16 *[28], *RASSF1A *[27]); metabolism and energy pathways (*GSTP1 *[9], *MDR1 *[29], *MTHFR *[25], *PTGS2 *[25], *TIMP3 *[25]); and regulation of nucleic acid metabolism (*ESR1 *[25], *ESR2 *[26], *FHIT*, *MGMT *[28], *RARβ2*). The primers and probe sequences studied have all been reported previously and can be found in the publications referenced after each gene. The primers and probe sequences used for the *FHIT *[GenBank: U76263] and *RARβ2 *[GenBank: NM_000965] were the following, respectively: sense, 5'-GGG CGC GGG TTT GGG TTT TTA C-3'; antisense, 5'-GAA ACA AAA ACC CAC CGC CCC G-3'; and probe, 6FAM-5'-AAC GAC GCC GAC CCC ACT AAA CTC C-3'-TAMRA and sense, 5'-GGG ATT AGA ATT TTT TAT GCG AGT TGT-3'; antisense, 5'-TAC CCC GAC GAT ACC CAA AC-3'; and probe, 6FAM-5'-TGT CGA GAA CGC GAG CGA TTC G-3'-TAMRA. To normalize for DNA input in each sample, a reference gene (*β-actin *[25]) was used.

Fluorescence based real-time PCR assays were carried out in a reaction volume of 20 μL, consisting of 16.6 mM ammonium sulfate; 67 mM trizma preset; 6.7 mM MgCl_2 _(2.5 mM for *p16*); 10 mM mercaptoethanol; 0.1% DMSO; 200 μM each of dATP, dCTP, dGTP, and dTTP; 600 nM of each primer; 0.4 μL of Rox dye; 200 nM of probe; 1 unit of platinum Taq polymerase (Invitrogen, Carlsbad, CA), and 2 μl of bisulfite-modified DNA as a template. PCR was performed in separate wells for each primer/probe set and each sample was run in triplicate. Additionally, multiple water blanks were used per plate, as a control for contamination (negative control). Leukocyte DNA collected from healthy individuals was methylated *in vitro *with excess *Sss*I CpG methylase (New England Biolabs Inc., Beverly, MA) to generate completely methylated DNA at all CpGs (positive control), and serial dilutions of this DNA after bisulfite conversion were used for constructing the calibration curve to quantify the amount of fully methylated alleles in each reaction. All amplifications were carried out in 96-well plates on an 7000 Sequence Detection System (Applied Biosystems, Foster City, C.A.), at 95°C for 2 min followed by 50 cycles of 95°C for 15 s, and 60°C for 1 min. For the *SFN *gene promoter QMSP assay, the reaction was carried out under the same conditions, except for the annealing temperature (62°C).

To determine the relative levels of methylated promoter DNA in each sample, the values obtained by QMSP analysis (mean quantity) for each target gene were divided by the respective values of the internal reference gene (*ACTB*). The ratio thus generated, which constitutes an index of the percentage of input copies of DNA that are fully methylated at the primer- and probe-binding sites, was then multiplied by 1000 for easier tabulation (methylation level = target gene/reference gene × 1000).

### Statistical analysis

The frequency of methylated samples, as well as the median and interquartile range of the methylation level for each target gene was determined. Values were analyzed using non-parametric tests, *i.e.*, the Kruskal-Wallis ANOVA, followed by the Mann-Whitney U test when appropriate. For multiple comparisons the Bonferroni method was used to adjust the P values. Results were considered statistically significant at the two-sided 5% significance level. Statistical analyses were carried out using a computer-assisted program (Statistica for Windows, version 6.0, StatSoft, Tulsa, OK).

## Results

### Clinical and pathological data

Relevant clinical and pathological characteristics of the patients included in this study are summarized in Table 1. As expected, males were more frequently diagnosed with renal cell tumor than females and ccRCC was the most frequent subtype. Most cases of RCC were confined to the organ and were classified as nuclear grade two or three.

**Table 1:** Clinical and pathological characteristics of patient population

Clinicopathological feature	
Patients, n	85
Gender, n (%)	
Male	52 (61.2)
Female	33 (38.8)
Age, yr, median (range)	61 (36–86)
Histologic subtype, n (%)	
Clear cell RCC	52 (61.2)
Papillary RCC	13 (15.2)
Chromophobe RCC	10 (11.8)
Oncocytoma	10 (11.8)
Pathologic stage*, n (%)	
T1	42 (56)
T2	15 (20)
T3	16 (21.4)
T4	2 (2.6)
Furhman grade, n (%)	
1	2 (2.7)
2	24 (32.0)
3	36 (48.0)
4	13 (17.3)

RCC, renal cell carcinoma, * only includes RCC cases

### QMSP in renal cell tumors and normal renal tissue

Using a QMSP assay, we examined the hypermethylation status of a panel of 18 cancer-related genes involved in several cancer pathways. The frequency and distribution of promoter methylation at each *locus *included in this panel are listed in Tables 2 and 3, respectively.

**Table 2:** Percentage and frequency of methylation [% (n)] of cancer-related genes in subtypes of renal cell carcinoma (RCC), oncocytoma, and morphologically normal renal tissue (NRT)

		Clear cell RCC	Papillary RCC	Chromophobe RCC	Oncocytoma	NRT
**Gene**	**Gene *loci***	% (n = 52)	% (n = 13)	% (n = 10)	% (n = 10)	% (n = 62)
*APC*	5q21-q22	19.2 (10)	23.1 (3)	0 (0)	0 (0)	8.1 (5)
*ARHI*	1p31	100 (52)	100 (13)	100 (10)	100 (10)	100 (62)
*CDH1*	16q22.1	82.7 (43)	69.2 (9)	20 (2)	30 (3)	87.1 (54)
*CTNNB1*	3p21	0 (0)	0 (0)	0 (0)	0 (0)	0 (0)
*SFN*	22q12.3	100 (52)	100 (13)	100 (10)	100 (10)	100 (62)
						
*ARF/p14*	9p21	9.6 (5)	7.7 (1)	10 (1)	20 (2)	27.4 (17)
*CDKN2A/p16*	9p21	0 (0)	0 (0)	0 (0)	0 (0)	0 (0)
*RASSF1A*	3p21.3	80.8 (42)	100 (13)	40 (4)	90 (9)	100 (62)
						
*GSTP1*	11q13	5.8 (3)	15.4 (2)	0 (0)	0 (0)	0 (0)
*MDR1*	7q21.1	86.5 (45)	84.6 (11)	80 (8)	90 (9)	96.8 (60)
*MTHFR*	1p36.3	100 (52)	100 (13)	100 (10)	100 (10)	100 (62)
*PTGS2*	1q25.2-q25.3	96.1 (50)	92.3 (12)	90 (9)	90 (9)	100 (62)
*TIMP3*	1p36.11	19.2 (10)	23.1 (3)	0 (0)	0 (0)	24.2 (15)
						
*ESR1*	6q25.1	67.3 (35)	76.9 (10)	60 (6)	80 (8)	77.4 (48)
*ESR2*	14q23.2	55.8 (29)	46.1 (6)	50 (5)	30 (3)	43.5 (27)
*FHIT*	3p14.2	51.9 (27)	53.8 (7)	50 (5)	50 (5)	69.3 (43)
*MGMT*	10q26	1.9 (1)	0 (0)	0 (0)	0 (0)	11.3 (7)
*RARβ2*	3p24	1.9 (1)	0 (0)	0 (0)	0 (0)	0 (0)

**Table 3:** Distribution of methylation levels of cancer-related genes in subtypes of renal cell carcinoma (RCC), oncocytoma, and morphologically normal renal tissue (NRT) [(target gene/*ACTB*) × 1000 expressed as median (interquartile range)]

**Gene**	Clear cell RCC	Papillary RCC	Chromophobe RCC	Oncocytoma	*p *value*	NRT
*APC*	0 (0-0)	0 (0-0)	0 (0-0)	0 (0-0)	ns	0 (0-0)
*ARHI*	942.6 (634.7–1746.6)	807.2 (623.3–1322.6)	198.6 (148.7–1575.5)	930.1 (641.5–1654.5)	ns	893.5 (574.2–1286.2)
*CDH1*	3.8 (0.5–12.3)	3.5 (0–19.3)	0 (0-0)	0 (0–1.1)	0.0007	2.3 (0.52–6.72)
*CTNNB1*	0 (0-0)	0 (0-0)	0 (0-0)	0 (0-0)	ns	0 (0-0)
*SFN*	1441.4 (947.3–2592.1)	1048.2 (848.3–1354.5)	1192.4 (687.9–1477.4)	1302.5 (738.6–2591.6)	ns	1088.4 (790.2–1497.1)
						
*ARF/p14*	0 (0-0)	0 (0-0)	0 (0-0)	0 (0-0)	ns	0 (0–0.04)
*CDKN2A/p16*	0 (0-0)	0 (0-0)	0 (0-0)	0 (0-0)	ns	0 (0-0)
*RASSF1A*	71.7 (4.7–265.5)	433.2 (236.4–966.0)	0 (0–2.7)	10.6 (2.2–27.5)	0.0001	59.6 (33.1–88.5)
						
*GSTP1*	0 (0-0)	0 (0-0)	0 (0-0)	0 (0-0)	ns	0 (0-0)
*MDR1*	25.3 (10.5–58.2)	15.5 (6.0–23.7)	33.1 (2.6–54.9)	33.5 (7.9–117.9)	ns	31.6 (16.6–41.9)
*MTHFR*	556.4 (236.9–965.9)	349.0 (181.6–561.9)	572.8 (340.3–887.0)	667.5 (634.4–1687.9)	ns	419.7 (305.2–614.3)
*PTGS2*	72.9 (34.1–222.5)	17.7 (9.3–43.2)	6.0 (3.9–49.7)	16.2 (5.3–28.9)	0.002	54.6 (34.4–90.8)
*TIMP3*	0 (0-0)	0 (0-0)	0 (0-0)	0 (0-0)	ns	0 (0-0)
						
*ESR1*	6.9 (0–31.3)	4.4 (2–10.9)	3.8 (0–19.1)	12.9 (7.7–76.5)	ns	15.0 (0.9–35.5)
*ESR2*	0.6 (0–1.9)	0 (0–0.4)	0.3 (0–2.7)	0 (0–0.5)	ns	0 (0–0.3)
*FHIT*	0.7 (0–5.6)	2.6 (0–47.0)	0.9 (0–4.0)	0.4 (0–6.6)	ns	2.0 (0–17.7)
*MGMT*	0 (0-0)	0 (0-0)	0 (0-0)	0 (0-0)	ns	0 (0-0)
*RARβ2*	0 (0-0)	0 (0-0)	0 (0-0)	0 (0-0)	ns	0 (0-0)

*Kruskall-Wallis one-way analysis of variance test among the four groups of renal cell tumors; IQR, interquartile range; ns, not significant

**Figure 1:**
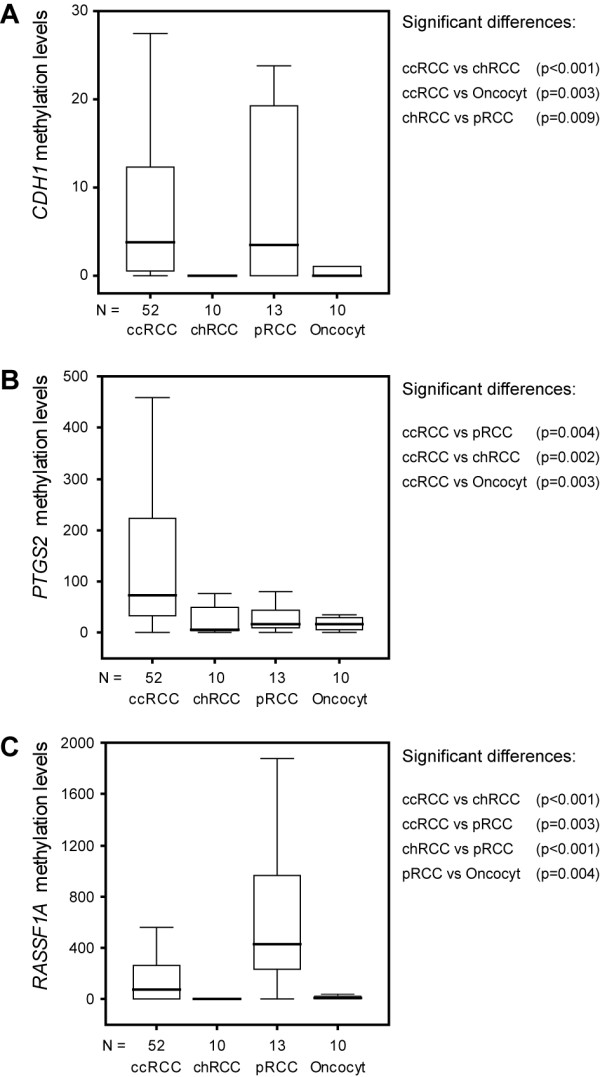
Methylation levels. Distribution of promoter methylation levels [(target gene/*ACTB*) × 1000] of (A) *CDH1*, (B) *PTGS2*, and (C) *RASSF1A *in clear cell (ccRCC), papillary (pRCC), and chromophobe (chRCC) renal carcinomas, and oncocytomas.

Promoter hypermethylation in at least one of the target genes was detected in all analyzed tissue samples, either tumor or normal renal tissue. *CTNNB1 *and *CDKN2A *promoter methylation was not found in any tissue sample. On the contrary, *ARHI*, *MTHFR*, and *SFN *promoter methylation was detectable in all tissue samples. Statistical analyses of distribution of methylation levels among the four tumor types disclosed significant differences for three genes, *i.e.*, *CDH1*, *PTGS2*, and *RASSF1A*. Pair-wise comparisons are shown and graphically illustrated in Fig. 1. Overall, chRCC and oncocytomas displayed a similar methylation profile, with low methylation levels in the three promoters. ccRCC are characterized by higher *PTGS2 *methylation levels, sharing with pRCC significant methylation at the *CDH1 *promoter. However, *RASSF1A *methylation stands as the most distinctive feature of pRCC in this epigenetic profile.

The 62 morphologically normal tissue samples matched in proportion those of the corresponding subtypes of RCC [39 (63%) from kidneys harboring ccRCC, 9 (14%) from pRCC, 6 (10%) from chRCC, and 8 (13%) from oncocytoma]. For all the target genes, methylation levels did not differ significantly among normal tissue samples stratified according with the paired renal cell tumor type. Moreover, no statistically significant differences were found between tumor and normal tissue samples concerning methylation levels at the *CDH1 *and *PTGS2 *promoters. However, *RASSF1A *methylation levels were significantly higher in pRCC than in normal tissue from all available cases (*p *= 0.002) and from kidneys harboring pRCC (*p *= 0.035).

After analyzing the methylation levels of the genes that were differentially methylated among the four tumor subtypes, empirical cutoff values were established to maximize the discriminative power of each gene (2.0 for *CDH1*, 80.0 for *PTGS2*, and 230.0 for *RASSF1A*). Validity estimates (sensitivity, specificity, positive and negative predictive values) were calculated and are depicted in Table 4.

**Table 4:** Validity estimates of *CDH1*, *PTGS2*, and *RASSF1A *hypermethylation for discrimination among subtypes of renal cell tumor

**Gene**	**Tumor subtypes discriminated**	**Sensitivity**	**Specificity**	**PPV**	**NPV**
***CDH1***	ccRCC & pRCC *vs. *chRCC & oncocytoma	61.3%	91.3%	95.0%	46.7%
***PTGS2***	ccRCC *vs. *pRCC, chRCC, & oncocytoma	46.2%	90.9%	88.9%	51.7%
***RASSF1A***	pRCC *vs. *ccRCC, chRCC, & oncocytoma	76.9%	75.0%	35.7%	94.7%

ccRCC, clear cell renal cell carcinoma; chRCC, chromophobe renal cell carcinoma; pRCC, papillary renal cell carcinoma; PPV, positive predictive value; NPV, negative predictive value

### Methylation profile and clinicopathologic parameters

Statistically significant associations between promoter methylation levels and standard clinicopathologic parameters were only found for pRCC. Specifically, *CDH1 *and *RASSF1A *promoter hypermethylation levels were inversely associated with tumor stage (*p *= 0.031) and nuclear grade (*p *= 0.022), respectively.

## Discussion

In this study we attempted to define a set of methylation markers that would allow for an accurate discrimination among the four most common types or renal cell tumor (ccRCC, pRCC, chRCC, and oncocytoma), as each type displays dissimilar clinical behavior and successful pre-operative cytological or histological assessment is restricted. Through gene promoter methylation profiling with QMSP, three genes were found to be differentially methylated in the four tumor types. In particular, higher *CDH1 *methylation levels were detected in ccRCC and pRCC, whereas high *RASSF1A *methylation levels were associated with pRCC and high *PTGS2 *methylation levels were characteristic of ccRCC. Remarkably, both chRCC and oncocytomas displayed low methylation levels at these three loci. Moreover, in pRCC, *CDH1 *and *RASSF1A *methylation levels correlated negatively with tumor stage and grade, respectively.

Overall, the frequency of promoter methylation found for the majority of genes was somewhat different compared with previous reports [16-20,30]. Differences in the patient populations, as well as in the method used for assessment of methylation, may account for these discrepancies, as QMSP is generally more sensitive and specific than conventional MSP, used in most of those earlier studies [24]. It is noteworthy that most previous publications included no or just a few chRCC, so our tumor series is more representative of the spectrum of renal cell tumors. Whereas we confirmed the high *RASSF1A *promoter methylation levels in pRCC previously reported by Gonzalgo and co-workers [17], we additionally found that *PTGS2 *promoter methylation as a likely candidate marker for ccRCC, something that might permit a more accurate detection of this tumor type in limited tissue or needle-aspirate samples.

An interesting finding of our study was the similar gene methylation profile of chRCC and oncocytoma, which has not been reported before to the best of our knowledge. This might be unexpected owing to the malignant character of the former and the benign behavior of the latter. However, both chRCC and oncocytoma share their origin from the distal nephron [31], a feature that might partially explain the epigenetic similarity. Moreover, these two tumor types might even look alike morphologically, as the eosinophilic variant of chRCC constitutes a differential diagnosis of oncocytoma [4]. Although we observed a tendency for higher *RASSF1A *methylation levels in chRCC than in oncocytomas, discriminating these two tumor types using methylation markers remains a challenging task which needs to be addressed in future studies in order to enable the critical distinction between a malignant and a benign tumor.

Clinicopathologic correlates with methylation levels have been previously reported by our research team in prostate cancer [11,12]. However, in the present study the correlations found for *CDH1 *and *RASSF1A *in pRCC are the in opposite direction of those described in prostate carcinomas, as higher *CDH1 *and *RASSF1A *methylation levels were associated with lower renal tumor stage and grade, respectively. Because cytogenetic complexity is associated with tumor progression in pRCC [4], the alternative acquisition of promoter methylation at *CDH1 *and *RASSF1A *loci might characterize a different subset of pRCC with less aggressive clinical behavior.

An important limitation of this study is the almost universal lack of statistically significant differences in methylation levels between morphologically normal and neoplastic renal tissues, with the notable exception of *RASSF1A *in pRCC. Gonzalgo and co-workers also found relatively high levels of methylation in normal renal tissue, collected from specimens harboring a renal neoplasm [17]. Interestingly, no significant differences in methylation levels were found among normal tissues procured from kidneys with different tumor types. Based on these findings, we are tempted to speculate that, at least in some cases, morphologically normal renal tissue might acquire aberrant methylation (eventually age-related) at some gene promoters owing to a "field-effect" phenomenon, with additional epigenetic or cytogenetic alterations then fostering tumor development. This hypothesis is consistent with previous reports of specific detection of renal malignancy in urine (in which normal renal epithelial cells are shed) using similar panels of methylation markers [16,20]. However, considering the main purpose of our study (*i.e.*, the development of methylation markers for detection and discrimination of renal cell neoplasms), the similar levels of promoter methylation in normal and neoplastic renal tissue constitutes a confounding variable which needs clarification, requiring the analysis of normal renal tissue from kidneys not harboring a neoplasm, which we are currently collecting.

## Conclusion

In conclusion, we found that a gene panel including *CDH1*, *PTGS2*, and *RASSF1A *which might contribute to a more accurate detection and discrimination of the common renal tumors. However, future studies addressing the discrimination between chRCC and oncocytoma, as well as the significance of aberrant methylation in morphologically normal renal tissue are required to allow the development of a clinically-useful, methylation-based test for renal tumor diagnosis.

## Abbreviations

MSP, methylation-specific PCR;

QMSP, quantitative methylation-specific PCR;

ccRCC, clear cell renal cell carcinoma;

pRCC, papillary renal cell carcinoma;

chRCC, chromophobe renal cell carcinoma;

*CDH1*, cadherin 1, type 1, E-cadherin (epithelial);

*PTGS2*, prostaglandin-endoperoxide synthase 2;

*RASSF1A*, Ras association domain family 1A;

RCC, renal cell carcinoma;

*ACTB*, beta actin;

NRT, normal renal tissue.

## Competing interests

The author(s) declare that they have no competing interests.

## Authors' contributions

VLC assisted in the study design, carried out the quantitative promoter methylation analyses, and drafted the manuscript; RH collected and classified all renal tumors, performed data analysis, and assisted in drafting the manuscript; FRR assisted with statistical analysis, and contributed to manuscript preparation; MP assisted with DNA extraction, and contributed to manuscript preparation. JO and FL contributed with clinical information for these patients and drafting the manuscript; MRT and CJ conceived the study, were responsible for its design and coordination, helped in the evaluation of the results and revised the manuscript critically for important intellectual content. All authors read and approved the final manuscript.

## Pre-publication history

The pre-publication history for this paper can be accessed here:



## References

[B1] Globocan database. http://www-dep.iarc.fr/.

[B2] Jemal A, Siegel R, Ward E, Murray T, Xu J, Smigal C, Thun MJ (2006). Cancer statistics, 2006. CA Cancer J Clin.

[B3] Pavlovich CP, Schmidt LS (2004). Searching for the hereditary causes of renal-cell carcinoma. Nat Rev Cancer.

[B4] Eble JN, Sauter G, Epstein JI, Sesterhenn IA (2004). Part 1: Tumours of the kidney. World health organization classification of tumours Pathology and genetics of tumours of the urinary system and male genital organs.

[B5] Young AN, Dale J, Yin-Goen Q, Harris WB, Petros JA, Datta MW, Wang MD, Marshall FF, Amin MB (2006). Current trends in molecular classification of adult renal tumors. Urology.

[B6] Lam JS, Leppert JT, Belldegrun AS, Figlin RA (2005). Novel approaches in the therapy of metastatic renal cell carcinoma. World J Urol.

[B7] Yang XJ, Sugimura J, Schafernak KT, Tretiakova MS, Han M, Vogelzang NJ, Furge K, Teh BT (2006). Classification of renal neoplasms based on molecular signatures. J Urol.

[B8] Jerónimo C, Henrique R, Sidransky D, Manel Esteller (2004). Uses of DNA methylation in cancer diagnosis and risk assessment. DNA methylation: approaches, methods, and applications.

[B9] Jerónimo C, Usadel H, Henrique R, Oliveira J, Lopes C, Nelson WG, Sidransky D (2001). Quantitation of *GSTP1 *methylation in non-neoplastic prostatic tissue and organ-confined prostate adenocarcinoma. J Natl Cancer Inst.

[B10] Harden SV, Sanderson H, Goodman SN, Partin AA, Walsh PC, Epstein JI, Sidransky D (2003). Quantitative *GSTP1 *methylation and the detection of prostate adenocarcinoma in sextant biopsies. J Natl Cancer Inst.

[B11] Jerónimo C, Henrique R, Hoque MO, Ribeiro FR, Oliveira J, Fonseca D, Teixeira MR, Lopes C, Sidransky D (2004). Quantitative RARβ2 hypermethylation: a promising prostate cancer marker. Clin Cancer Res.

[B12] Jerónimo C, Henrique R, Hoque MO, Mambo E, Ribeiro FR, Varzim G, Oliveira J, Teixeira MR, Lopes C, Sidransky D (2004). A quantitative promoter methylation profile of prostate cancer. Clin Cancer Res.

[B13] Bastian PJ, Palapattu GS, Lin X, Yegnasubramanian S, Mangold LA, Trock B, Eisenberger MA, Partin AW, Nelson WG (2005). Preoperative serum DNA *GSTP1 *CpG island hypermethylation and the risk of early prostate-specific antigen recurrence following radical prostatectomy. Clin Cancer Res.

[B14] Bastian PJ, Ellinger J, Wellmann A, Wernert N, Heukamp LC, Muller SC, von Ruecker A (2005). Diagnostic and prognostic information in prostate cancer with the help of a small set of hypermethylated gene loci. Clin Cancer Res.

[B15] Enokida H, Shiina H, Urakami S, Igawa M, Ogishima T, Li LC, Kawahara M, Nakagawa M, Kane CJ, Carroll PR, Dahiya R (2005). Multigene methylation analysis for detection and staging of prostate cancer. Clin Cancer Res.

[B16] Hoque MO, Begum S, Topaloglu O, Jeronimo C, Mambo E, Westra WH, Califano JA, Sidransky D (2004). Quantitative detection of promoter hypermethylation of multiple genes in the tumor, urine, and serum DNA of patients with renal cancer. Cancer Res.

[B17] Gonzalgo ML, Yegnasubramanian S, Yan G, Rogers CG, Nicol TL, Nelson WG, Pavlovich CP (2004). Molecular profiling and classification of sporadic renal cell carcinoma by quantitative methylation analysis. Clin Cancer Res.

[B18] de Caceres II, Dulaimi E, Hoffman AM, Al-Saleem T, Uzzo RG, Cairns P (2006). Identification of novel target genes by an epigenetic reactivation screen of renal cancer. Cancer Res.

[B19] Battagli C, Uzzo RG, Dulaimi E, de Caceres II, Krassenstein R, Al-Saleem T, Greenberg RE, Cairns P (2003). Promoter hypermethylation of tumor suppressor genes in urine from kidney cancer patients. Cancer Res.

[B20] Dulaimi E, de Caceres II, Uzzo RG, Al-Saleem T, Greenberg RE, Polascik TJ, Babb JS, Grizzle WE, Cairns P (2004). Promoter hypermethylation profile of kidney cancer. Clin Cancer Res.

[B21] Fuhrman SA, Lasky LC, Limas C (1982). Prognostic significance of morphologic parameters in renal cell carcinoma. Am J Surg Pathol.

[B22] Pearson H, Stirling D (2003). DNA extraction from tissue. Methods Mol Biol.

[B23] Clark SJ, Harrison J, Paul CL, Frommer M (1994). High sensitivity mapping of methylated cytosines. Nucleic Acids Res.

[B24] Eads CA, Danenberg KD, Kawakami K, Saltz LB, Blake C, Shibata D, Danenberg PV, Laird PW (2000). MethyLight: a high-throughput assay to measure DNA methylation. Nucleic Acids Res.

[B25] Eads CA, Lord RV, Wickramasinghe K, Long TI, Kurumboor SK, Bernstein L, Peters JH, DeMeester SR, DeMeester TR, Skinner KA, Laird PW (2001). Epigenetic patterns in the progression of esophageal adenocarcinoma. Cancer Res.

[B26] Müller HM, Widschwendter A, Fiegl H, Ivarsson L, Goebel G, Perkmann E, Marth C, Widschwendter M (2003). DNA methylation in serum of breast cancer patients: an independent prognostic marker. Cancer Res.

[B27] Lehmann U, Länger F, Feist H, Glöckner S, Hasemeier B, Kreipe H (2002). Quantitative assessment of promoter hypermethylation during breast cancer development. Am J Pathol.

[B28] Harden SV, Tokumaru Y, Westra WH, Goodman S, Ahrendt SA, Yang SC, Sidransky D (2003). Gene promoter hypermethylation in tumors and lymph nodes of stage I lung cancer patients. Clin Cancer Res.

[B29] Yegnasubramanian S, Kowalski J, Gonzalgo ML, Zahurak M, Piantadosi S, Walsh PC, Bova GS, de Marzo AM, Isaacs WB, Nelson WG (2004). Hypermethylation of CpG islands in primary and metastatic human prostate cancer. Cancer Res.

[B30] Tokinaga K, Okuda H, Nomura A, Ashida S, Furihata M, Shuin T (2004). Hypermethylation of the RASSF1A tumor suppressor gene in Japanese clear cell renal cell carcinoma. Oncol Rep.

[B31] Polascik TJ, Bostwick DG, Cairns P (2002). Molecular genetics and histopathologic features of adult distal nephron tumors. Urology.

